# Synergistic effects on mesenchymal stem cell-based cartilage regeneration by chondrogenic preconditioning and mechanical stimulation

**DOI:** 10.1186/s13287-017-0672-5

**Published:** 2017-10-03

**Authors:** Sien Lin, Wayne Yuk Wai Lee, Qian Feng, Liangliang Xu, Bin Wang, Gene Chi Wai Man, Yuanfeng Chen, Xiaohua Jiang, Liming Bian, Liao Cui, Bo Wei, Gang Li

**Affiliations:** 1 Department of Orthopaedic Center, Affiliated Hospital of Guangdong Medical University, Guangdong Medical University, Zhanjiang, China; 2 Department of Pharmacology and Guangdong Key Laboratory for Research and Development of Natural Drugs, Guangdong Medical University, Zhanjiang, China; 3 Department of Orthopaedics & Traumatology, Faculty of Medicine, The Chinese University of Hong Kong, Prince of Wales Hospital, Hong Kong, China; 4 The CUHK-ACC Space Medicine Centre on Health Maintenance of Musculoskeletal System, The Chinese University of Hong Kong Shenzhen Research Institute, Shenzhen, China; 5 Department of Mechanical and Automation Engineering, the Chinese University of Hong Kong, Hong Kong, China; 6 Key Laboratory for Regenerative Medicine of Ministry of Education, School of Biomedical Sciences, Faculty of Medicine, The Chinese University of Hong Kong, Hong Kong, China

**Keywords:** Tissue engineering, Mesenchymal stem cells, Chondrogenesis, Mechanotransduction, Cartilage diseases

## Abstract

**Background:**

Mesenchymal stem cells (MSCs) hold promising translational potential in cartilage regeneration. However, the efficacy of MSC-based tissue engineering is not satisfactory in the treatment of cartilage defect because of the inevitable cellular functional changes during ex vivo cell expansion. How to maintain the chondrogenic capacity of MSCs to improve their therapeutic outcomes remains an outstanding question.

**Methods:**

Bone marrow-derived MSCs were firstly primed in chondrogenic induction medium which was then replaced with normal growth medium to attain the manipulated cells (M-MSCs). Methacrylated hyaluronic acid (MeHA) was synthesized as a scaffold to encapsulate the cells. The MSC- or M-MSC-laden constructs were treated with dynamic compressive loading (DL) in a bioreactor or with free loading (FL) for 14 days. Afterwards, the constructs were implanted in nude mice or rat models of osteochondral defects to test their efficiency in cartilage regeneration or repair.

**Results:**

Data showed that the resulting M-MSCs exhibited superior chondrogenic differentiation potential and survivability compared with untreated MSCs. More importantly, we found that DL significantly promoted neocartilage formation in the MeHA hydrogel encapsulated with M-MSCs after 30 days of implantation in nude mice. Furthermore, the constructs laden with M-MSCs after DL for 14 days significantly enhanced cartilage healing in a rat model of osteochondral defect.

**Conclusions:**

Findings from this study highlight the importance of maintaining chondrogenic potential of MSCs by in-vitro chondrogenic preconditioning and a synergistic effect of mechanical stimulation in cartilage engineering, which may shed light on the stem cell-based tissue engineering for cartilage repair.

**Electronic supplementary material:**

The online version of this article (doi:10.1186/s13287-017-0672-5) contains supplementary material, which is available to authorized users.

## Background

Articular cartilage damage resulting from a sudden injury or wear and tear could predispose to development of traumatic osteoarthritis (OA). Patients suffering from OA have pain and stiffness at the affected joints which reduces their work efficiency, daily activities, and quality of life. It is estimated that 900,000 cases of knee injuries happen annually in the United States, and post-traumatic OA accounts for 12% of all cases of OA [1]. Pre-existing anterior cruciate ligament injury, with or without a concomitant meniscus injury, is another risk for OA development [2]. As the etiology and pathogenesis of OA remain elusive, the current medications are solely prescribed to relieve the symptoms rather than ameliorating the status of cartilage deterioration. Conventional surgical procedures including arthroscopic debridement [3], bone marrow stimulation [4, 5], and autograft or allograft mosaicplasty [6] are used to stimulate cartilage repair, but none of these treatments can prevent the affected cartilage from progressive destruction [7].

Current innovative approaches for articular cartilage repair are functional tissue engineering strategies combined mesenchymal stem cells (MSCs), biomimetic scaffold, growth factors, and mechanical stimuli [8, 9]. Regenerative medicine using MSCs provides a potential solution to the regeneration ability by forming a cartilage-like tissue without immunogenicity [10–12]. However, differentiation and proliferation capacity of MSCs are reported to be age-dependent [7, 13, 14] which could restrict the efficacy of MSCs. Furthermore, MSCs propagated in a culture dish could undergo uncontrolled changes to their phenotype after long-term ex-vivo culture [7]. Thus, they deviate markedly from their original nature and function [15]. How to maintain the chondrogenic property of MSCs without compromising the number of final cell products is one of the major challenges in tissue engineering for the treatment of cartilage lesions. Apart from genetic manipulation, our previous studies demonstrated that environmental preconditioning was a reliable method to improve the properties of stem cells and promote lineage differentiation commitment, and finally enhance the therapeutic effects [16–18]. Most recently, we found that cell viability and the chondrogenic potential of MSCs were significantly enhanced after preconditioning in chondrogenic medium before cell expansion [19]. Even more, the manipulated MSCs (M-MSCs) presented superior therapeutic outcomes in a surgically induced OA animal model compared with untreated MSCs [19].

Aside from the MSCs, a biocompatible scaffold is essential to support the chondrogenic differentiation ability of MSCs to form engineered cartilage. Various materials have been reported as scaffolds. Hyaluronic acid (HA) is among those drawing special attention because of its biocompatibility and physiological role as a key building material for healthy cartilage [20, 21]. HA can be modified to photo-crosslink into three dimensional (3D) hydrogels that aid chondrogenesis of MSCs [20]. The superior mechanical stiffness, network porosity, and permeability of HA have also been shown to have a positive impact on the differentiation of encapsulated MSCs, distribution of newly synthesized cartilage matrix, and nutrition transportation [22, 23]. Our previous research indicated that dynamic compressive loading (DL) was also of great help for promoting chondrogenesis of MSCs and inhibiting subsequent hypertrophy [8, 22]. In order to develop a novel strategy to overcome the major hurdles faced by current MSC-based cartilage tissue engineering, we hypothesized that a combination of chondrogenic medium preconditioning and DL may enhance the efficiency of MSC-based cartilage tissue engineering and promote cartilage repair in an osteochondral defect model.

## Methods

### Study design

**Fig. 1:**
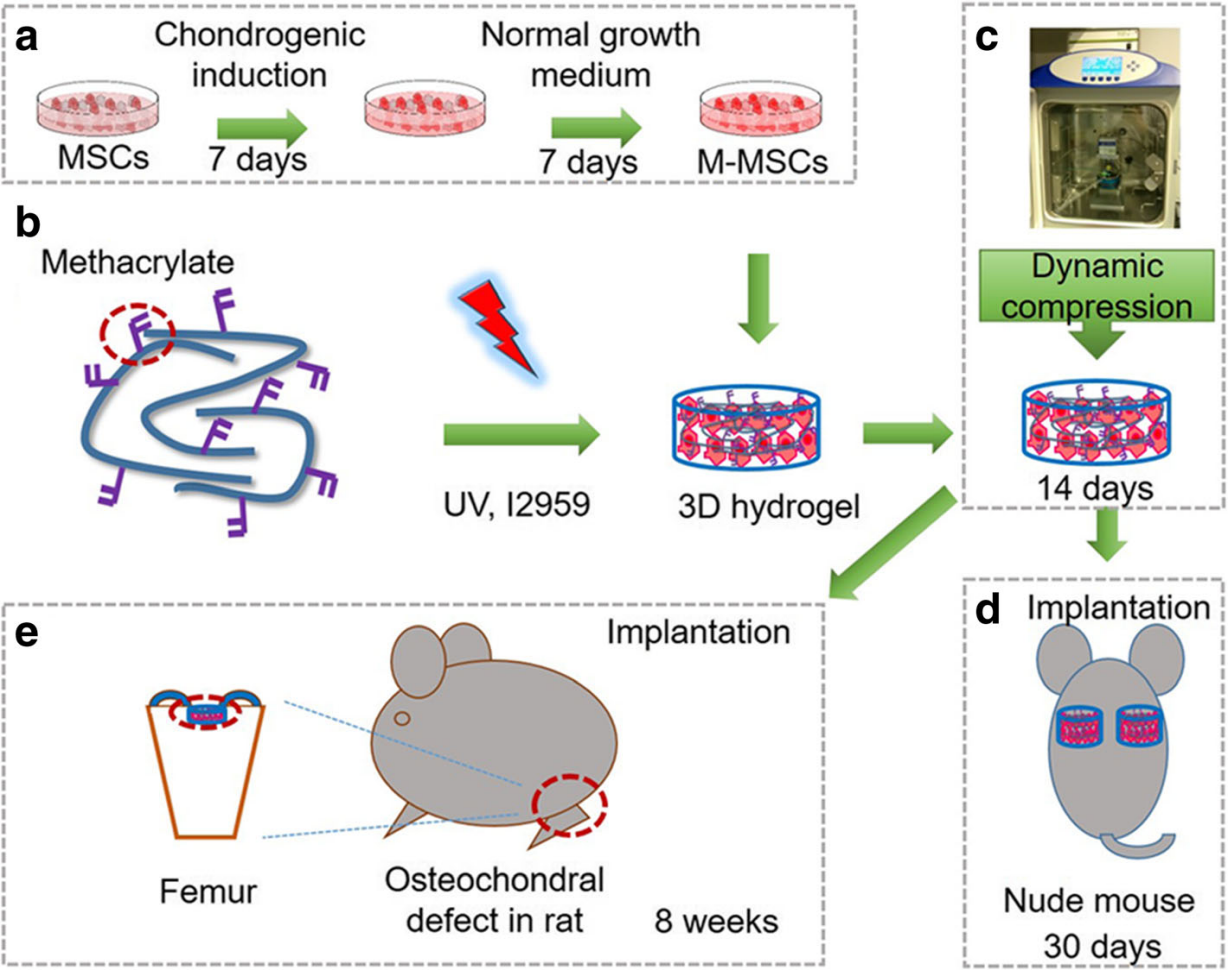
Schematic diagram of the study design. **a** Procedure for deriving manipulated mesenchymal stem cells (*M-MSCs*) via chondrogenic preconditioning. To obtain chondrogenic preconditioned M-MSCs, MSCs were firstly incubated with chondrogenic induction medium for 7 days which was then followed by basal growth medium for another 7 days. **b** UV-initiated crosslinking of methacrylated hyaluronic acid (MeHA) laden with cells (20 × 10^6^ cells/mL). **c** Compressive loading to the cell-seeded MeHA hydrogel in a bioreactor. M-MSCs or untreated MSCs photoencapsulated in MeHA hydrogel were subjected to free loading or dynamic loading in a bioreactor for 14 days. **d** Subcutaneous implantation in nude mice. After 14 days of culture in a bioreactor, the constructs were subcutaneously implanted in nude mice for 30 days. **e** Implantation in osteochondral defect in rats. After 14 days of culture in a bioreactor, the constructs were implanted in an osteochondral defect in rats. Samples were harvested after 8 weeks

This study aimed to determine the efficiency of a novel tissue engineering strategy, i.e., combined stepwise preconditioning and mechanical loading, in cartilage regeneration or repair. Figure 1 shows the schematic diagram of this study. MSCs were firstly primed in chondrogenic induction medium (CIM) which was then replaced with normal growth medium to attain the manipulated cells (M-MSCs) (Fig. 1a). Methacrylated HA (MeHA) was used to form a scaffold to encapsulate the cells (Fig. 1b). The constructs were then laden with MSCs or M-MSCs and were treated with dynamic compressive loading (DL) in a bioreactor (Fig. 1c). After the designated loading period, the constructs were tested for their efficiency in cartilage formation or repair in nude mice (Fig. 1d) or an osteochondral defect rat model (Fig. 1e).

### Cell culture and preconditioning in vitro

Rat MSCs were harvested from the bone marrow of adult male outbred green fluorescent protein (GFP) Sprague-Dawley rats (SD-Tg (CAG-EGFP) Cz-004Osb) as previously reported [18]. In brief, the bone marrow was flushed out from the bone cavity and subject to density gradient centrifugation to obtain the mononuclear cells (MNCs). The MNCs were cultured in α-minimal essential medium (α-MEM) containing 10% fetal bovine serum and 100 U/mL penicillin (all from Invitrogen) at 37 °C with 5% CO_2_. The isolated MNCs were plated at an optimized low density (500 cells/cm^2^) for the isolation of MSCs and cultured at 37 °C with 5% CO_2_ to form colonies; medium was changed every 3 days. Cells at passage 3–5 were used for all experiments.

Firstly, MSCs were incubated with chondrogenic induction medium (CIM) containing Dulbecco’s modified Eagle’s medium (DMEM) supplemented with 10 ng/mL transforming growth factor-β1 (PerproTech), 10^–7^ M dexamethasone, 50 mg/mL ascorbate-2-phosphate, 40 mg/mL proline, 100 mg/mL pyruvate (all from Sigma-Aldrich), and 1:100 diluted (ITS + Premix) (Becton Dickinson) in culture flask for 7 days. CIM was then replaced with basal growth medium (α-MEM) and allowed to grow for another 7 days. MSCs cultured in basal culture medium for 14 days served as control (MSCs).

### Chondrogenic differentiation

8 × 10^5^ cells (M-MSCs or MSCs) were centrifuged to form pellets and cultured in CIM; medium was changed every 3 days. Total RNA was extracted with Trizol® (Invitrogen, Carlsbad, CA) at day 7 for quantitative real-time polymerase chain reaction (qRT-PCR). At day 21, the cell pellets were fixed with 4% buffered formaldehyde (Sigma-Aldrich) for further histological analysis.

### Hydrogel preparation

MeHA was synthesized as previously reported [8]. Briefly, methacrylic anhydride (94%, MW: 154.17, Sigma-Aldrich) was added to a solution of 1% (w/v) HA (sodium hyaluronate powder, MW: ~ 74 kDa, Lifecore) in deionized water, adjusted to a pH of 8 with 5 N NaOH, and reacted on ice for 24 h. The macromer solution was purified via dialysis (MW cutoff 6–8 kDa) against deionized water for a minimum of 48 h with repeated changes of water. The final product was obtained by lyophilization and stored at –20 °C in powder form prior to use. The final macromer products were confirmed by ^1^H nuclear magnetic resonance (NMR) to have a methacrylation level of ~ 29%. Lyophilized macromers were dissolved in phosphate-buffered saline (PBS) containing 0.05 wt% of the photoinitiator 2-methyl-1-[4-(hydroxyethoxy)phenyl]-2-methyl-1-propanone (I2959, Ciba) to allow for ultraviolet (UV)-mediated polymerization.

After preconditioning in chondrogenic medium for 7 days and basal medium for another 7 days, M-MSCs (20 × 10^6^ cells/mL) were then photo-encapsulated with UV light (wavelength 360 nm; intensity 1.2 Mw/cm^2^) in 2% MeHA hydrogel disks (diameter 5 mm, 2.0-mm thickness). Untreated MSCs incubated in basal medium were also photo-encapsulated in MeHA hydrogel as controls. Cell survivability was determined by alamarBlue assay immediately after UV exposure. Cell viability was also tested on these 3D cell culture samples by calcein AM (live) and ethidium bromide (dead) after UV exposure. Images were taken under confocal laser scanning microscope (Leica TCS SP8). Histology (Alcian blue staining) and qRT-PCR were also performed to investigate the chondrogenic potential on 3D cell culture samples after 14 days.

### Dynamic compressive loading

After 1 day of in-vitro culture in chondrogenic medium, the constructs were subjected to free loading (FL) or dynamic compressive loading (DL) with the CartiGen Bioreactor System (Instron, Norwood, MA) in chondrogenic medium. The compressive loading protocol was applied as described previously [8, 24]. Briefly, the loading protocol consisted of a 10% peak compressive sinusoidal strain at 1 Hz frequency, superimposed on a 5% compressive tare strain. Loading was carried out in unconfined compression via impermeable loading pistons for 4 h per day and 5 days per week. DL was carried out in a humidified incubator for 14 days. FL control cultures were positioned adjacent to the loading device during this period.

### Ectopic transplantation of hydrogel in nude mice

After preconditioning in chondrogenic medium for 7 days and basal medium for another 7 days, M-MSCs (20 million/mL) photo-encapsulated in MeHA hydrogel were subjected to FL or DL in the bioreactor for 14 days as described above. Then M-MSC- or MSC-seeded (20 million/mL) MeHA hydrogel constructs preincubated under DL or FL conditions (*n* = 12 per group) were implanted subcutaneously into male nude mice (*n* = 12). Four subcutaneous pockets were prepared on the dorsum of each nude mouse and received implants under anesthesia. After 30 days of implantation, fresh samples were harvested for the mechanical test (*n* = 8 constructs) and q-RT PCR (*n* = 4 constructs). After the mechanical test, these samples were cut into two equal parts and subjected to histology and immunohistochemistry (*n* = 8 halves), or biochemical analysis (*n* = 8 halves).

### Mechanical test

Samples removed from nude mice were subjected to the mechanical test in uniaxial, unconfined compression using the MACH-1™ Mechanical Testing System (Biomomentum, Laval, QC, Canada) as previously described [25]. Briefly, sample diameter and thickness were firstly determined by electronic digital caliper (Maplin Electronics, South Yorkshire, UK). They were equilibrated by creep to a tare load of 2 g by an impermeable loading plate in a loading chamber filled with PBS and, from this offset, stress relaxation tests were performed with a single compression ramp at a speed of 10%/min until reaching 20% strain. The equilibrium Young’s modulus (Eγ) was determined by the equilibrium load obtained after 1000 s of relaxation under unconfined compression at 20% strain using the MACH-1 Analysis software (Biomomentum, Laval). All given strains and strain rates were referenced to the initial 2 mm thickness of the specimens. Each sample was subsequently deformed to a specific compressive strain level (25–80%) at a strain rate of 100%/min, and the peak loads were recorded.

### Biochemical analysis

After the mechanical test, the constructs (*n* = 8 halves) were weighed wet, lyophilized, reweighed dry, and digested in 0.5 mg/mL Proteinase-K (Fisher Scientific) at 56 °C for 16 h. The PicoGreen assay (Invitrogen, Molecular Probes) was used to quantify the DNA content of the constructs, with Lambda phage DNA (0–1 mg/mL) as the standard [26]. For each sample, the masses of both the entire gel and the half gel used for the DNA assay were measured. The total amount of DNA per sample was calculated by scaling the amount of DNA detected in the half gel by a weight ratio (total weight/half weight). The glycosaminoglycan (GAG) content was measured using the dimethylmethylene blue (Sigma-Aldrich, St. Louis, MO) dye-binding assay, with shark chondroitin sulfate (0–50 mg mg/mL) as the standard [27]. The overall collagen content was assessed by measuring the orthohydroxyproline (OHP) content via dimethylaminobenzaldehyde and chloramine T assay. The collagen content was calculated by assuming a 1:7.5 OHP-to-collagen mass ratio [28]. The collagen and GAG contents were normalized to the disk wet weight.

### Gene expression

Total RNA extracted from cell pellets (*n* = 3 replicates from each donors) or hydrogel constructs (*n* = 4 per group) were used to perform qRT-PCR. Complementary DNA quantification was performed in duplicate by qRT-PCR using Step-One-Plus Real Time PCR Systems (Applied Biosystems) and normalized against GAPDH expression. Primer sequences were determined through established GenBank sequences (Table 1).

**Table 1:** Sequences of primers for quantitative real time polymerase chain reaction

Gene name	Forward primer sequence (5’ to 3’)	Reverse primer sequence (5’ to 3’)
GAPDH	AGCCCAGAACATCATCCCTG	CACCACCTTCTTGATGTCATC
Sox9	AGAGCGTTGCTCGGAACTGT	TCCTGGACCGAAACTGGTAAA
Acan	TTGTGACTCTGCGGGTCATC	GTCCCTAGGAGGGCCTTCAG
Col 2a1	AACCCAAAGGACCCAAATAC	CCGGACTGTGAGGTTAGGAT
Col 10a1	ATATCCTGGGGATCCAGGTC	TCCAGGTTCACCTCTTGGAC
MMP13	AGGCCTTCAGAAAAGCCTTC	GAGCTGCTTGTCCAGGTTTC

### Implantation of constructs into osteochondral defects in rats

After in-vitro culture in the bioreactor for 14 days, the constructs laden with MSCs (20 million/mL) or M-MSCs (20 million/mL) preincubated under DL or FL conditions were implanted into the osteochondral defects in the femoral trochlear groove of SD rats under anesthesia as previously described [29]. Briefly, 4-month-old SD rats (*n* = 50) were anesthetized with ketamine and xylazine and the right knee joints were exposed through a medial parapatellar approach after shaving and disinfection. The patella was dislocated laterally and the knee placed in full flexion. Then a defect (diameter 1.5 mm, and 1.5 mm in depth) was created in the center of the groove using a dental drill. All debris was removed from the defect with a curette and irrigation. Depending on the experimental group, the defects were implanted with MeHA hydrogel loading with MSCs (FL), M-MSCs (FL), MSCs (DL), or M-MSCs (DL), with 10 rats in each group in a press-fit way. The patella was physically relocated, and the joint capsule, subcutaneous tissue, and skin were closed with sutures. The right femur was collected under anesthesia by an overdose of pentobarbital after 8 weeks. The left femur was collected as the intact control. Femoral samples were fixed in 10% buffered formalin and then decalcified in 10% buffered ethylenediaminetetraacetic acid (EDTA; pH 7.4, Sigma-Aldrich) followed by the Safranin O and Fast Green (both from Sigma-Aldrich) and immunohistochemical staining with type II collagen (Col II) or GFP antibody.

### Histological scoring of osteochondral defect healing

Histological sections from the anterior to posterior regions of each defect (a total of 16 or 20 images per group) were blindly scored by three independent researchers based on a previously established scoring system (Table 2) [30, 31]. Briefly, sections were scored for the extent of cartilage repair based on five criteria: cell morphology, matrix staining, surface regularity, thickness of cartilage, and integration of donor with host adjacent cartilage.

**Table 2:** Cartilage repair score according to Wakitani et al. [31]

Parameters	Score
Cell morphology	
Hyaline cartilage	0
Mostly hyaline cartilage	1
Mostly fibrocartilage	2
Mostly noncartilage	3
Noncartilage only	4
Matrix staining	
Normal (compared with host adjacent cartilage)	0
Slightly reduced	1
Markedly reduced	2
No metachromatic stain	3
Surface regularity (total smooth area compared with entire area of cartilage defect)	
Smooth (>3/4)	0
Moderate (>1/2–3/4)	1
Irregular (1/4–1/2)	2
Severely irregular (<1/4)	3
Thickness of cartilage (compared with that of surrounding cartilage)	
> 2/3	0
1/3–2/3	1
< 1/3	2
Integration of donor with host adjacent cartilage	
Both edges integrated	0
One end integrated	1
Neither edge integrated	2

### Immunohistochemistry

The expressions of Col II and GFP in the hydrogel constructs (*n* = 8 halves per group) or rat samples (*n* = 10 per group) were detected by immunohistochemistry. The samples were incubated with primary antibodies against Col II (1:5; Abcam) or GFP (1:200; Santa Cruz) and subsequently with horseradish peroxidase (HRP)-conjugated secondary antibody (1:200; Santa Cruz). Primary antibody was replaced with blocking solution in the negative controls. The sections were examined under light microscopy. ImageJ (NIH) was introduced to analyze the Col II- or GFP-positive area in the constructs or defect sites.

### Statistical analysis

All the quantitative data are presented as mean ± standard deviation (SD). After checking for a normal distribution using the Kolmogorov-Smirnov test, all parameters were analyzed by analysis of variance (ANOVA) and post-hoc Tukey’s honest significant difference (HSD). For histological scoring, nonparametric Mann-Whitney *U* test was used for comparisons between groups. The statistical analysis was calculated by SPSS (version 16; SPSS Inc, Chicago, IL) and the level of significance was set at *P* < 0.05.

## Results

### Chondrogenic capacity of M-MSCs

**Fig. 2:**
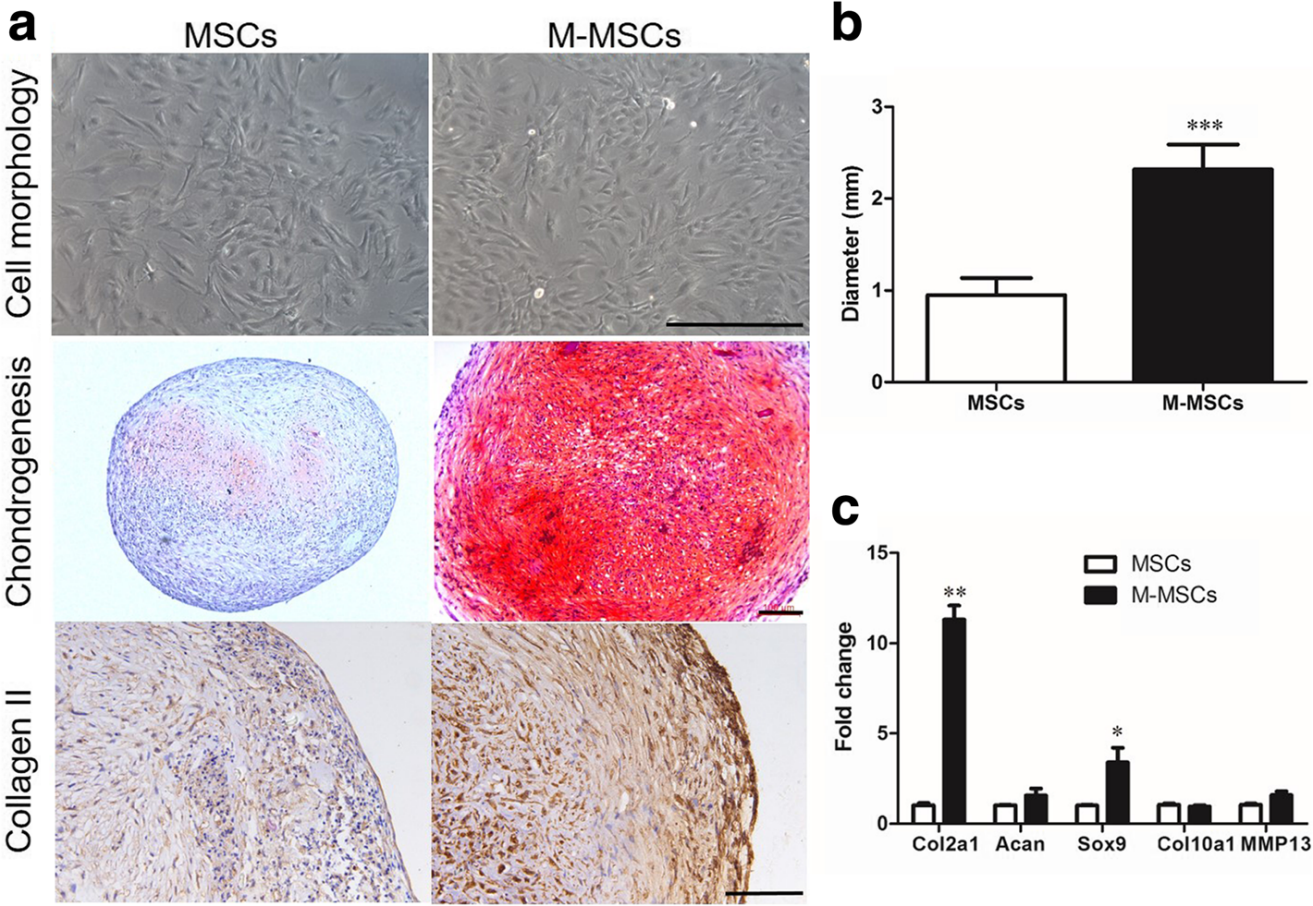
Cell morphology and chondrogenic property of M-MSCs in vitro. **a** Cell morphology in two-dimensional culture and staining results of Safranin O or type II collagen (Col II) after chondrogenic differentiation for 21 days in three-dimensional cell pellets. **b** Size of pellets (diameter). **c** Expression levels of chondrogenic markers (Col2a1, Acan, and Sox9) and hypertrophic markers (Col10a1 and MMP13) determined by qRT-PCR assays. Values represent means ± SD (*n* = 3 donors with three replicates per donor). *P < 0.05, **P < 0.01, ***P < 0.001, vs. MSCs. *Scale bar* = 100 μm. *M-MSC* manipulated mesenchymal stem cell, *MSC* mesenchymal stem cell

The cell morphology and chondrogenic potential of M-MSCs and untreated MSCs are shown in Fig. 2. After chondrogenic preconditioning, M-MSCs regained a spindle-like shape and similar morphology to the untreated MSCs (Fig. 2a). M-MSCs exhibited higher chondrogenic capacity compared with untreated MSCs (Fig. 2a and c) which was consistent with our recent report [19]. Results illustrated that large amounts of GAG and Col II, a much larger pellet size (*P* < 0.001), and upregulated expressions of Col2a1 (*P* < 0.01), Acan (*P* > 0.05), and Sox9 (*P* < 0.05) were found in the M-MSC pellets compared with untreated MSCs (Fig. 2a and b).

**Fig. 3:**
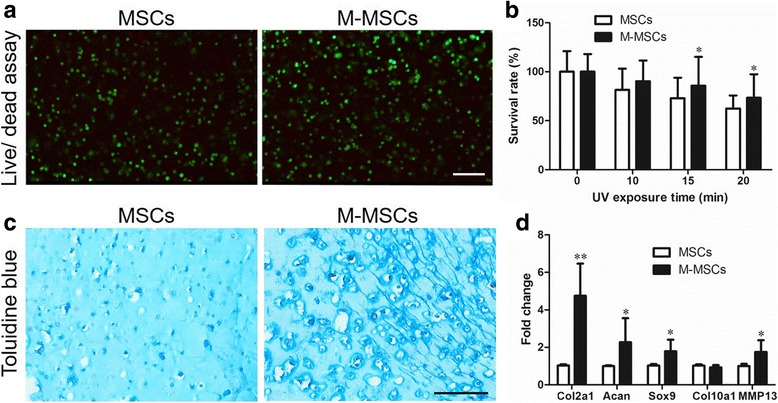
Cell viability and chondrogenic property in MeHA hydrogel. Assays were performed after 14 days of in-vitro culture. **a** Fluorescent images of cell viability as measured by live/dead cell assay after UV-initiated crosslinking. **b** Quantitative results of cell viability as measured by alamarBlue assays. **c** Results of Alcian Blue staining of constructs after 14 days of culture. **d** Gene expression in three-dimensional cell culture samples after 14 days of culture. Values represent means ± SD (*n* = 4 replicates). *P < 0.05, **P < 0.01, ***P < 0.001, vs. MSCs. *Scale bar* = 100 μm. *M-MSC* manipulated mesenchymal stem cell, *MSC* mesenchymal stem cell, *UV* ultraviolet

Fluorescent images of live/dead cells in MeHA hydrogel after UV exposure were used to verify the cell viability (Fig. 3a). Quantitative data of alarmarBlue assay showed a higher cell survival rate in M-MSC-laden constructs after 15 min (85.7% in M-MSCs vs*.* 73.0% in MSCs, *P* < 0.05) or 20 min (73.3% in M-MSCs vs*.* 62.5% in MSCs, *P* < 0.05) of UV exposure (Fig. 3b). Moreover, results of histology and qRT-PCR also indicated higher chondrogenic capacity in the MeHA hydrogel encapsulated with M-MSCs (Fig. 3c and d).

### Cartilaginous tissue regeneration in nude mice

**Fig. 4:**
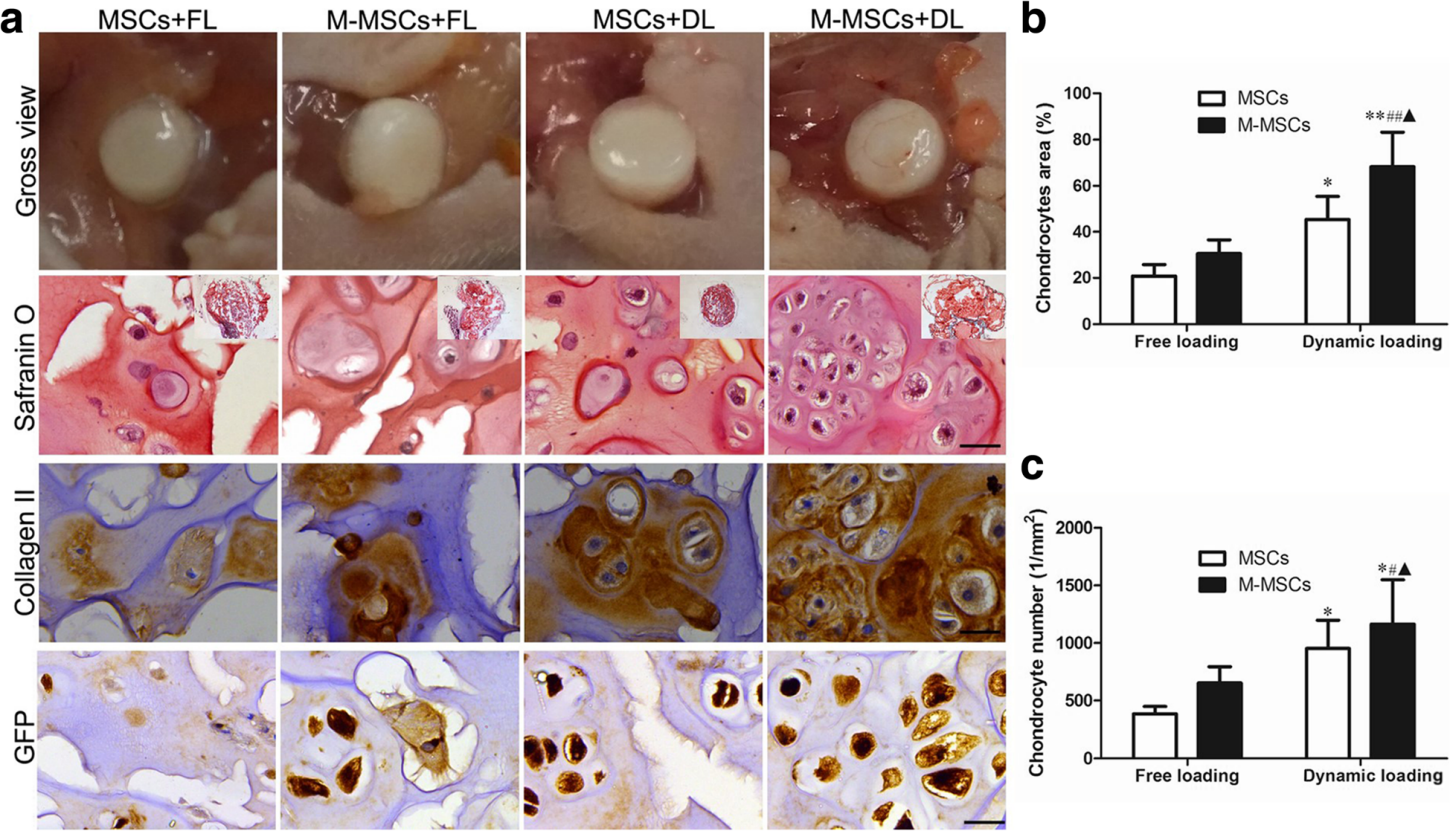
Histological results of tissue engineered cartilage. Assays were performed after 14 days of chondrogenic differentiation under free loading (*FL*) or dynamic compressive loading (*DL*) conditions in the bioreactor and 30 days of ectopic implantation in nude mice. **a** Representative gross view and histology of the resulting constructs. Sections stained with Safranin O or immunohistochemically stained with type II collagen and green fluorescent protein (*GFP*). *Scale bar* = 10 μm. **b** Semi-quantitative data of cartilage area. **c** Semi-quantitative data of chondrocyte number in the constructs. Values represent means ± SD (*n* = 8). *P < 0.05, **P < 0.01, vs. MSCs + FL; ^#^ *P* < 0.05, ^##^ *P* < 0.01, vs. M-MSCs + FL; ^▲^ *P* < 0.05 vs. MSCs + DL. *M-MSC* manipulated mesenchymal stem cell, *MSC* mesenchymal stem cell

After dynamic compressive loading for 14 days, the constructs laden with M-MSCs or untreated MSCs and the loading-free controls were transplanted subcutaneously into nude mice for a further 30 days. At the endpoint, cartilage-like samples were found in all the groups (Fig. 4a). Safranin O staining revealed that the constructs laden with M-MSCs after dynamic compressive loading showed distinct advantages in the formation of cartilaginous tissue (Fig. 4a) compared with the constructs laden with MSCs under the FL condition, as illustrated by the increasing chondrocyte area (68.3% vs*.* 20.8%, *P* < 0.01) and chondrocyte number (384/mm^2^ vs*.* 1164/mm^2^, *P* < 0.05) (Fig. 4b and c).

**Fig. 5:**
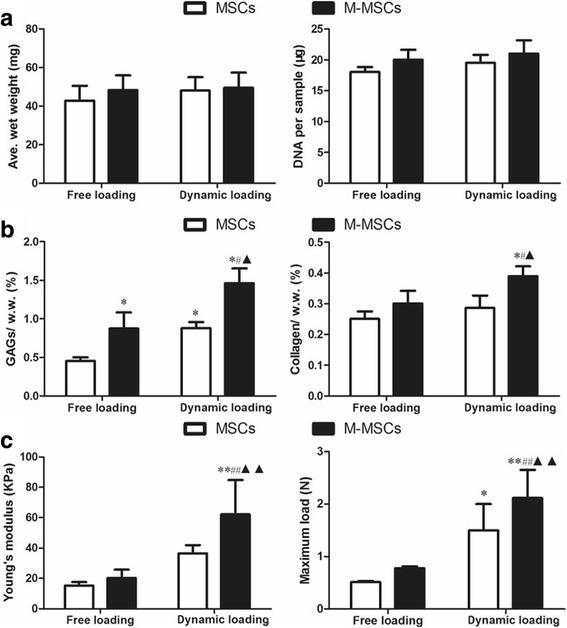
Matrix deposition and mechanical properties of the tissue engineered cartilage. Assays were performed after 14 days of chondrogenic differentiation under free loading (FL) or dynamic compressive loading (DL) conditions and 30 days of ectopic implantation in nude mice. **a** Average wet weight (*Ave. wet weight*) and DNA content per sample. **b** Cartilaginous matrix (glycosaminoglycans (*GAGs*) and collagen) deposition of the constructs, normalized to wet weight (*w.w.*). **c** Mechanical properties (Young’s modulus and ultimate load) of the constructs laden with M-MSCs or untreated MSCs. Values represent means ± SD (*n* = 8). *P < 0.05, **P < 0.01, vs. MSCs + FL; ^#^ *P* < 0.05, ^##^ *P* < 0.01, vs. M-MSCs + FL; ^▲^ *P* < 0.05, vs. MSCs + DL. *M-MSC* manipulated mesenchymal stem cell, *MSC* mesenchymal stem cell

The cartilage-specific matrix components inside the constructs were determined after enzymatic digestion. The GAG and total collagen content after normalization to their wet weight (w.w.) were used to indicate the quality of the constructs [22, 32]. Our findings showed that there was no significant difference in the average wet weight and contents of DNA among the groups (Fig. 5a). However, we found that dynamic compressive loading significantly increased the content of GAG and collagen in the constructs laden with M-MSCs by 62.3% (*P* < 0.05) and 29.6% (*P* < 0.05) compared with their FL counterparts, respectively (Fig. 5b). Moreover, dynamic compressive loading further increased the matrix content of GAG and collagen in the M-MSC group by 66.1% (*P* < 0.05) and 35.9% (*P* < 0.05) when compared with those of MSCs (Fig. 5b). Overall, the GAG and collagen content in the M-MSC group after dynamic compressive loading were increased by 221.1% (*P* < 0.05) and 55.4% (*P* < 0.05), respectively, when compared with the MSC + FL group, indicating a synergistic effect of chondrogenic preconditioning and dynamic compressive loading (Fig. 5b).

A mechanical test was performed on the fresh samples. As demonstrated in Fig. 5c, a significantly higher Young’s modulus (+70.2%, *P* < 0.01) and maximum load (+41.1%, *P* < 0.01) were observed in the constructs laden with M-MSCs when compared with MSCs after dynamic loading. Dynamic loading also markedly increased the Young’s modulus and maximum load in the constructs laden with M-MSCs by 207.1% (*P* < 0.01) and 172.8% (*P* < 0.01) compared with the FL counterparts (Fig. 5c).

**Fig. 6:**
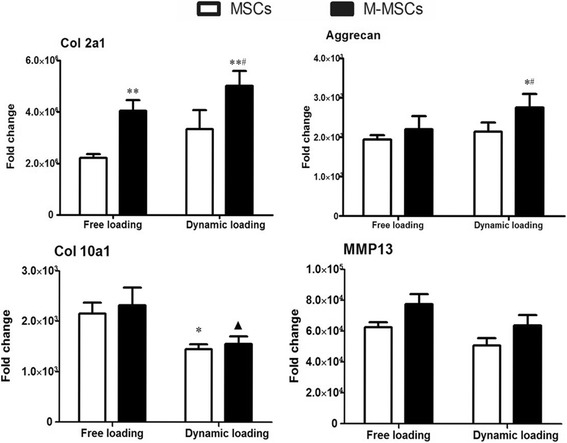
Gene expression in the tissue-engineered cartilage. Assays were performed after 14 days of chondrogenic differentiation under free loading (FL) or dynamic compressive loading (DL) conditions and 30 days of ectopic implantation in nude mice. Expression of chondrogenic markers (Col2a1 and Aggrecan) and hypertrophic markers (Col 10a1 and MP13) in the tissue-engineered cartilage were measured. Values represents means ± SD (*n* = 4). *P < 0.05, **P < 0.01, vs. MSCs + FL; ^#^ *P* < 0.05, vs. M-MSCs + FL; ^▲^ *P* < 0.05, vs. MSCs + DL. *M-MSC* manipulated mesenchymal stem cell, *MSC* mesenchymal stem cell

Chondrogenic-related gene expression profiles in the same constructs were determined using qRT-PCR. Our results showed a significantly higher transcription level of mRNA coding for type II collagen (Col2a1, *P* < 0.01) and aggrecan (Acan, *P* < 0.05) in M-MSCs after dynamic loading when compared with the FL counterparts (Fig. 6). More importantly, the hypertrophic marker type X collagen (Col10a1, *P* < 0.05) was downregulated in both M-MSCs and MSCs after dynamic compressive loading (Fig. 6).

### Healing outcomes of cartilage defects

**Fig. 7:**
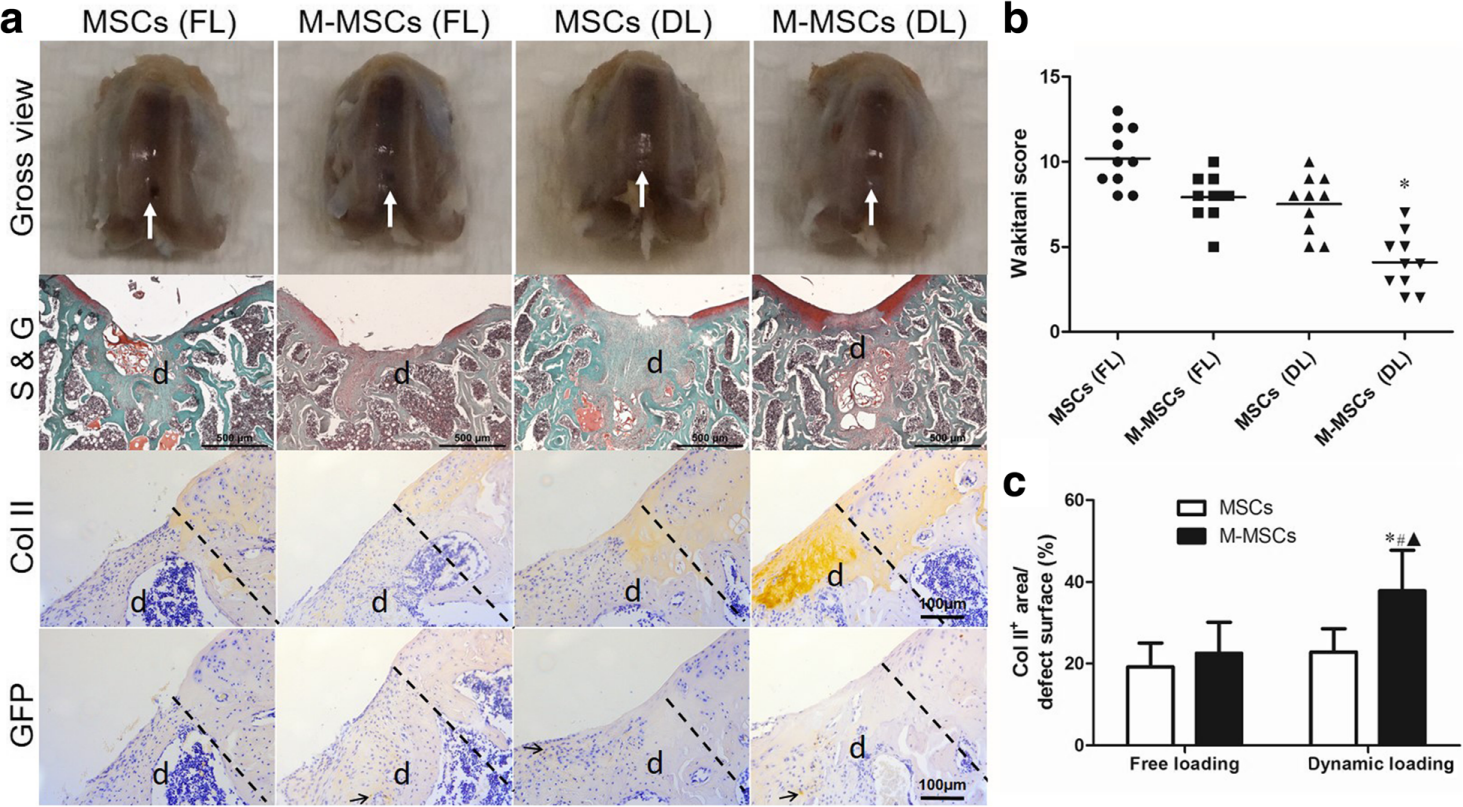
Repair effect of engineered cartilage in an osteochondral defect rat model after 2 months of implantation. Cell-seeded MeHA hydrogels were incubated in chondrogenic medium and pretreated with free loading (*FL*) or dynamic loading (*DL*) in the bioreactor for 14 days before implantation. **a** Representative gross view and histology of distal femoral samples stained with Safranin O and fast green (*S & G*) or immunohistochemically stained with type II collagen (*Col II*) and green fluorescent protein (*GFP*). *White arrows* point to the defect sites. *Black arrows* point to the GFP-positive area. *Dashed lines* indicate the interface of native tissue and the defect sites (*d*). **b** Semi-quantitative data (Wakitani score) after treatments. **c** Semi-quantitative data of the Col II-positive area in the defect area. Values represents means ± SD (*n* = 10). *P < 0.05, vs. MSCs + FL. *M-MSC* manipulated mesenchymal stem cell, *MSC* mesenchymal stem cell

An osteochondral defect rat model was used to evaluate the therapeutic outcome of the constructs. After 8 weeks of implantation, histological examinations were performed to show matrix production, and type II collagen and GFP expression (Fig. 7). In general, the vast majority of the samples in the MSCs + FL group contained only a thin layer of fibrous tissue and residual HA hydrogel on the surface of the defect (Fig. 7a). The MSCs + DL and M-MSCs + FL groups contained large amounts of fibrocartilage (Fig. 7a). However, the M-MSCs + DL group often had hyaline-like cartilage with much more expression of type II collagen rather than fibrocartilage at the defect surface (Fig. 7a and c). In scoring the morphology of the newly formed surface tissue, it was observed that the M-MSCs + DL group had higher quality surface tissue compared to the other groups (Fig. 7b and c). In addition, we found only a small number of GFP-positive cells remaining at the defect area (less than 2%) in all the animals, suggesting that the majority of the GFP-positive MSCs were eliminated after 2 months of implantation in SD rats (Additional file 1: Figure S1). Furthermore, we observed the formation of bony tissue surrounding the residual hydrogel in the subchondral bone area, indicating good compatibility and an osteoconductive property of the MeHA material (Additional file 2: Figure S2).

## Discussion

This is the first study to demonstrate the synergistic effect of chondrogenic preconditioning and dynamic compressive loading on MSC-mediated cartilage repair for a focal osteochondral defect. This present study, together with our previous studies [17–19], suggest a feasible manipulation by a defined medium which could enhance the lineage-specific differentiation ability and cell survivability via an epigenetic modification of pluripotent genes, such as demethylation of Nanog and Oct4, during the stepwise preconditioning [18, 19].

Dynamic compressive loading has been demonstrated to enhance the chondrogenic ability of MSCs, although the underlying mechanism is not clear. A previous study revealed that as long as 70 days of dynamic compressive loading increased the mechanical properties, as well as the GAG and collagen contents, of HA hydrogel constructs [8]. In this study, the chondrogenic preconditioning prior to dynamic loading could result in a significant improvement in cell viability and chondrogenic capacity of MSCs laden in HA constructs over 14 days. Results of the histological analysis in nude mice also revealed that more chondrocytes expressing intense type II collagen and GFP existed in the M-MSCs + DL group compared with that of the MSCs + DL group, indicating that the production and distribution of cartilage matrix were significantly enhanced by the chondrogenic preconditioning. Our results also revealed that gene expression levels of hypertrophic markers such as type X collagen (Col10a1) and MMP13 were not significantly increased in M-MSCs vs*.* MSCs after in-vitro culture and in-vivo implantation, indicating that hypertrophy could not be accelerated by the chondrogenic preconditioning. Moreover, gene expression levels of type X collagen and MMP13 were downregulated in the constructs seeded with MSCs or M-MSCs after dynamic loading, indicating an inhibition effect of compressive loading on hypertrophy during chondrogenic differentiation, consistent with our previous finding [8]. In summary, we have demonstrated a simple and feasible method of producing engineered cartilage with enhanced chondrogenic potential and cell viability by a combination of chondrogenic preconditioning and dynamic loading, representing a novel approach for cartilage tissue engineering. Although we have no evidence to demonstrate the underlying mechanism in this study, dynamic compressive loading in our study may increase nutrient transport into the hydrogel constructs, leading to enhancements in production and distribution of cartilage matrix produced by MSCs [33, 34]. Previous research revealed that Indian hedgehog (IHH) and parathyroid hormone-related protein (PTHrP) were two key factors in the regulation of the hypertrophic differentiation process of chondrocytes [35]. These factors are regarded as mechanically responsive molecules in downregulating hypertrophic markers of MSCs during mechanical loading [35, 36],

In the osteochondral defect model, M-MSC-laden constructs with dynamic loading prior to implantation showed better therapeutic outcome as indicated by a higher expression level of cartilage matrix and enhanced integration into the native tissues at the defect site. Previous studies described that MeHA hydrogels provide a stable three-dimensional environment which is favorable to chondrogenesis of human MSCs and neocartilage formation [8]. A previous study also demonstrated that MeHA hydrogel functionalized by N-cadherin provided a supportive niche microenvironment for osteogenesis of human MSCs [37]. In this study, the residual MeHA hydrogel was surrounded with bony tissue in the subchondral bone area, indicating that this material was biocompatible and osteoconductive. Furthermore, there was no obvious sign of chondrocyte aggregation within the defect site in all the groups, although chondrocyte aggregation and associated intense type II collagen expression was found in the nude mice.

This discrepancy is likely due to different microenvironments, in particular the enriched immune signals inside the defect site. Unlike nude mice, the SD rat is immunocompetent, with functional macrophages, B cells, and T cells which are responsive to foreign antigens. Although MSCs are well recognized to be immunocompatible and safe when they are applied in allotransplantation, it is also reported that chondrogenic differentiation could increase their immunogenicity by upregulating B7 molecules [38]. Furthermore, the rat model suffers from limitations due to joint size, thin cartilage, and the potential for improved intrinsic repair because of life-long open growth plates [39]. In order to draw a more convincing conclusion, a larger animal model and a population of MSCs with lower immunogenicity should be taken into consideration in a follow-up study.

## Conclusions

Taken together, we conclude here that chondrogenic potential and cell survivability could be significantly enhanced by chondrogenic preconditioning and dynamic compressive loading. The resulting M-MSC-laden HA constructs showed superior efficacy in cartilage regeneration in nude mice and were more effective for cartilage healing in osteochondral defect models. Findings from this study highlight the importance of maintaining chondrogenic potential of MSCs by chondrogenic preconditioning and a synergistic effect of mechanical stimulation in cartilage engineering, which may shed light on the advancement of stem cell-based therapy for cartilage repair.

## Additional files

Additional file 1: **Figure S1.** Semi-quantitative results of GFP-positive cell ratio in the defect area. GFP-positive cell ratio = GFP-positive cells in the defect area/all the cells in defect area × 100%. (TIF 619 kb)

Additional file 2: **Figure S2.** Representative histological images of material-tissue reaction. Assays were performed after 2 months of implantation of MeHA hydrogel. Samples were stained with hematoxylin and eosin (H&E) (A) or Safranin O and Fast Green (B). (TIF 3188 kb)

## Abbreviations

α-MEM: α-Minimal essential medium
CIM: Chondrogenic induction medium
Col II: Type 2 collagen
DL: Dynamic compressive loading
FL: Free loading
GAG: Glycosaminoglycan
GFP: Green fluorescent protein
HA: Hyaluronic acid
MeHA: Methacrylated hyaluronic acid
M-MSC: Manipulated mesenchymal stem cell
MNC: Mononuclear cell
MSC: Mesenchymal stem cell
NMR: Nuclear magnetic resonance
OA: Osteoarthritis
OHP: Orthohydroxyproline
PBS: Phosphate-buffered saline
qRT-PCR: Quantitative real-time polymerase chain reaction
UV: Ultraviolet
