# Supplementary figures and images for: Synergistic effects on mesenchymal stem cell-based cartilage regeneration by chondrogenic preconditioning and mechanical stimulation
Source: Stem Cell Res Ther. 2017 Oct 3;8:221. doi: 10.1186/s13287-017-0672-5 (PMC5627486; doi:10.1186/s13287-017-0672-5)

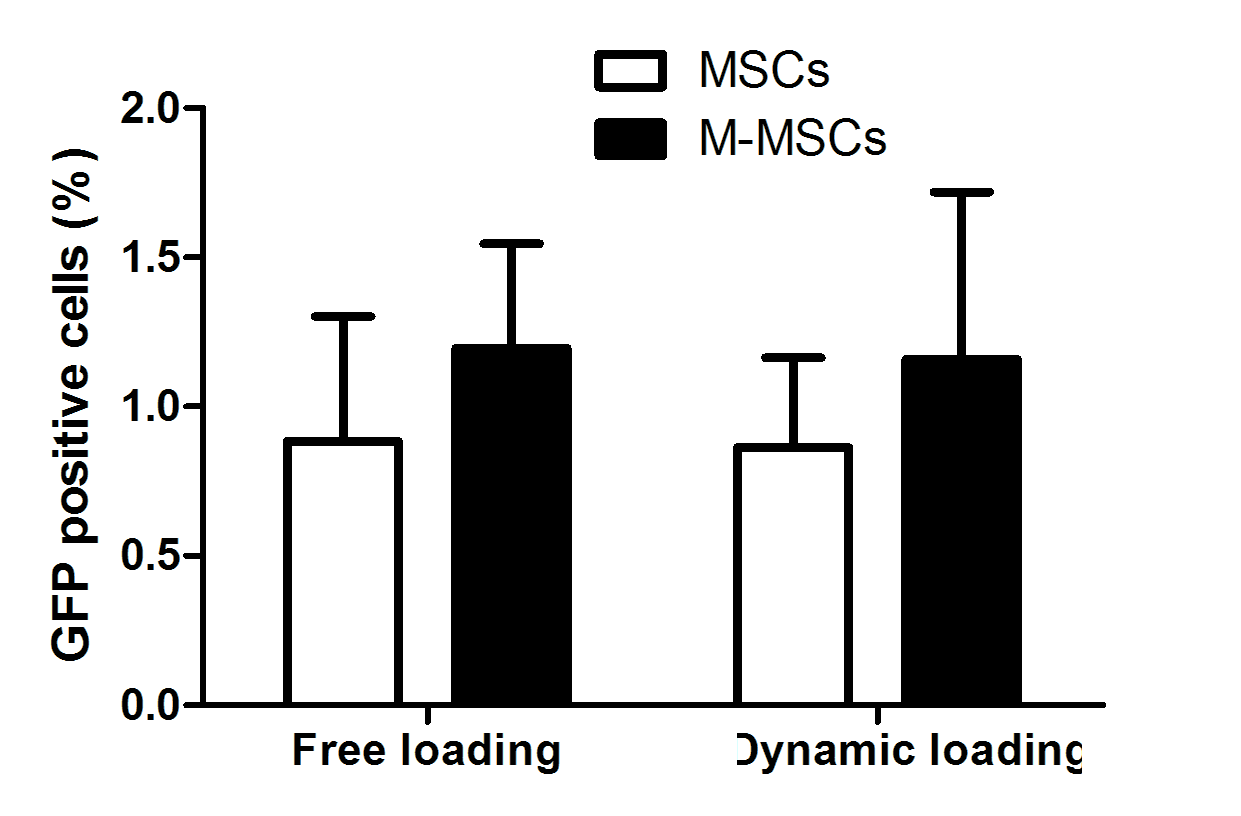

Supplement: Supplementary file 1 — Figure S1. Semi-quantitative results of GFP-positive cell ratio in the defect area. GFP-positive cell ratio = GFP-positive cells in the defect area/all the cells in defect area × 100%. (TIF 619 kb) [file 13287_2017_672_MOESM1_ESM.tif]

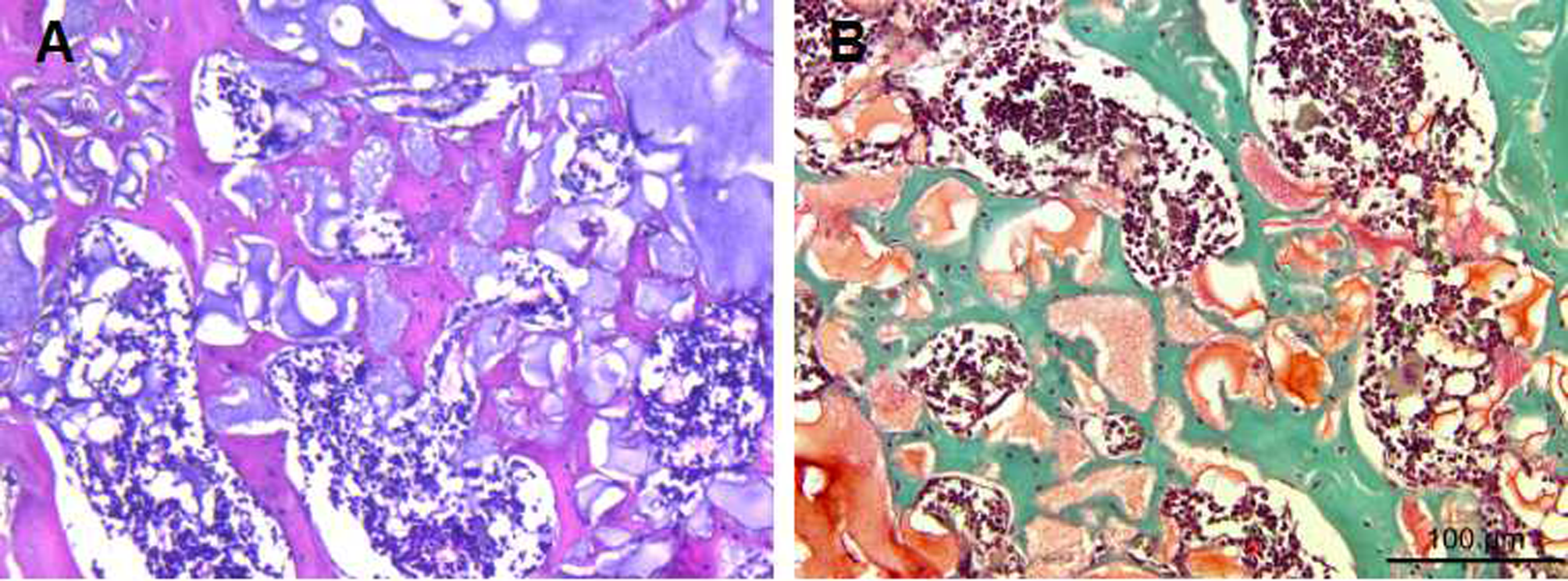

Supplement: Supplementary file 2 — Figure S2. Representative histological images of material-tissue reaction. Assays were performed after 2 months of implantation of MeHA hydrogel. Samples were stained with hematoxylin and eosin (H&E) (A) or Safranin O and Fast Green (B). (TIF 3188 kb) [file 13287_2017_672_MOESM2_ESM.tif]
